# T cell‐expressed *Ift88* is required for proper thymocyte differentiation in mice

**DOI:** 10.14814/phy2.70120

**Published:** 2024-11-19

**Authors:** Sarah J. Miller, Nancy M. Gonzalez, Morgan E. Smith, Mandy J. Croyle, Bradley K. Yoder, Kurt A. Zimmerman

**Affiliations:** ^1^ Department of Internal Medicine, Division of Nephrology and Hypertension University of Oklahoma Health Sciences Center Oklahoma City Oklahoma USA; ^2^ Department of Cell, Developmental, and Integrative Biology University of Alabama at Birmingham Birmingham Alabama USA

**Keywords:** thymocyte development, thymus

## Abstract

Intraflagellar transport protein 88 (*Ift88*) is required for the formation of cilia in the thymus and non‐ciliary dependent functions including T cell immune synapse formation. To test the role of *Ift88* in T cell development, we performed flow cytometry analysis on thymus and spleen tissue isolated from mice lacking *Ift88* in thymic epithelial cells (TECs) or T cells. Analyses indicated that TEC *Ift88* deletion had no impact on thymic T cell development and minimal impact on splenic T cells. Analysis of T cells in *CaggCre* ^*ERT2+*^ *Ift88* ^tm1Bky^mTmG mice indicate that approximately half of DN1 thymocytes are *Ift88* deficient 3 weeks post‐tamoxifen induction; *Ift88* loss did not impact T cell development at the DN2‐DN4 stage or the CD4+/CD8+ double‐positive (DP) thymocyte stage. However, survival of *Ift88* deficient T cells was significantly reduced at the single‐positive (SP) thymocyte stage, as was the number of CD4+ and CD8+ T cells in spleen and kidney. Despite preferential survival of *Ift88*‐proficient cells, the total number of T cells the in spleen and kidney was minimally impacted by *Ift88* loss. These data suggest *Ift88* is required for differentiation of DP thymocytes into SP thymocytes and that *Ift88* proficient T cells can compensate for deficient cells to fill the open niche.

## 1 INTRODUCTION

Intraflagellar transport protein 88 (*Ift88*) is involved in multiple cellular processes including cilia formation, oriented cell division, and cell migration (Boehlke et al., 2015; Borovina & Ciruna, 2013; Hildebrandt et al., 2011). In addition, *Ift88* has been reported to be present in the immune synapse (Finetti et al., 2009), although the importance of *Ift88* in T cell development, differentiation, and function is not well understood. Congenital deletion of *Ift88* results in embryonic lethality (Gilley et al., 2014); therefore, a conditional allele was generated to analyze the function of *Ift88* in adult mice. This was accomplished by crossing conditional *Ift88* ^tm1Bky^ mice (*Ift88* ^*f/f*^) with mice containing either a thymic epithelial cell‐specific cre recombinase (*Foxn1* ^*cre*^) or a globally expressed, tamoxifen inducible cre recombinase (*CaggCre* ^*ERT2*^). Importantly, our data indicate that *CaggCre* ^*ERT2*^ is active in ~40% of T cells as they enter the thymus (Sharma et al., 2013).

Lymphoid progenitors from the bone marrow seed the thymus (Shortman & Wu, 1996) via the corticomedullary junction (Dzhagalov & Phee, 2012). During the process of maturation, thymocytes migrate from the corticomedullary junction to the cortex and eventually on to the medulla (Dzhagalov & Phee, 2012). The initial steps in thymocyte development occur in the cortex where double‐negative (DN) T cells lacking CD4+ and CD8+ expression undergo beta selection, a process that eliminates thymocytes with gross defects in TCR‐β rearrangement (Dudley et al., 1994). As thymocytes undergo beta selection, they must pass through four DN stages (DN1–DN4), marked by changes in expression of CD44 and CD25. Passage through DN1 (CD44 + CD25−), DN2 (CD44+, CD25+), DN3 (CD44−, CD25+), and DN4 (CD44−, CD25−) stages results in the removal of most non‐functional T cells. Cells that undergo successful TCR‐β rearrangement upregulate CD4 and CD8, becoming double‐positive (DP) thymocytes that undergo positive and negative selection in the cortex (Starr et al., 2003). This process results in the rescue of cells from programmed cell death that are non‐reactive (positive selection) and the removal of cells that are autoreactive (negative selection) (Carpenter & Bosselut, 2010; Starr et al., 2003). Finally, the DP thymocytes become MHC class restricted, determining their singly positive (SP) lineage commitment (CD4+ T helper or CD8+ cytotoxic T cells).

*Ift88* has been reported to exist as part of an immune complex with *Ift20* and *Ift57 (*Finetti et al., 2009). Deletion of *Ift20* using a CD4^cre^ did not affect the number of T cells in the thymus, spleen, blood, or lymph nodes (Vivar et al., 2016; Yuan et al., 2014). In contrast, deletion of *Ift20* at earlier stages of T cell development using an Lck^cre^ resulted in reduced CD4+ and CD8+ SP thymocytes (Yuan et al., 2014). Although deletion of *Ift20* using the CD4^cre^ did not affect the number of T cells during homeostasis, TCR signaling and Zap70 phosphorylation were reduced in OTI and OTII T cells following stimulation with ovalbumin (OVA) peptides (Vivar et al., 2016). This was associated with an impaired proliferative capacity and reduced ability to produce IFNγ following T cell activation. Despite the extensive data linking *Ift20* to T cell function, the importance of *Ift88* in T cell development and function is less appreciated. Data from Finetti and colleagues show that *Ift88* is abundantly expressed in Jurkat T cells, although surprisingly, little IFT88 protein was observed in human peripheral blood lymphocytes, isolated human T cells, or in whole spleen or lymph tissue isolated from mice (Finetti et al., 2009). In addition to its described role in immune synapse formation, *Ift88* and cilia have also been reported in the thymus (Bautista et al., 2021; Kutomi et al., 2022; Michelson et al., 2022). An earlier study from Kutomi and colleagues reported cilia were present on thymic epithelial cells and that loss of thymic epithelial cell‐expressed *Ift88* resulted in increased CD4+ and CD8+ T cells in old mice although no effects were observed in newborn or 3–4 week old mice (Kutomi et al., 2022; Wang et al., 2020).

To test the importance of *Ift88* in thymocyte development and maturation, we performed flow cytometry analysis of thymocytes isolated from Foxn1^cre^ *Ift88* ^f/f^ and CaggCre^ERT2^ *Ift88* ^f/f^ mTmG mice 3 weeks post tamoxifen induction. Our data indicate that loss of *Ift88* in thymic epithelial cells (Foxn1^cre^ *Ift88* ^f/f^) did not impact T cell development in the thymus and had minimal impact on CD4+ and CD8+ T cells in the spleen eight‐week‐old mice. In contrast, loss of *Ift88* in T cells using the globally‐expressed CaggCre^ERT2^ prevented DP thymocytes from transitioning into the SP thymocyte stage. The reduced number of *Ift88*‐deficient SP CD4+ and CD8+ T cells was also observed in the spleen and kidney. Despite the drastic reduction in the ratio of *Ift88*‐deficient to *Ift88* wild‐type SP CD4+ and CD8+ T cells in the thymus, kidney, and spleen, the overall number of CD4+ and CD8+ T cells was not different. These studies suggest that T cells with normal expression of *Ift88* compensate for the loss of their *Ift88*‐deficient counterparts.

## 2 RESULTS

### 2.1 Loss of *Ift88* in thymic epithelial cells does not impact T cell development

**FIGURE 1:**
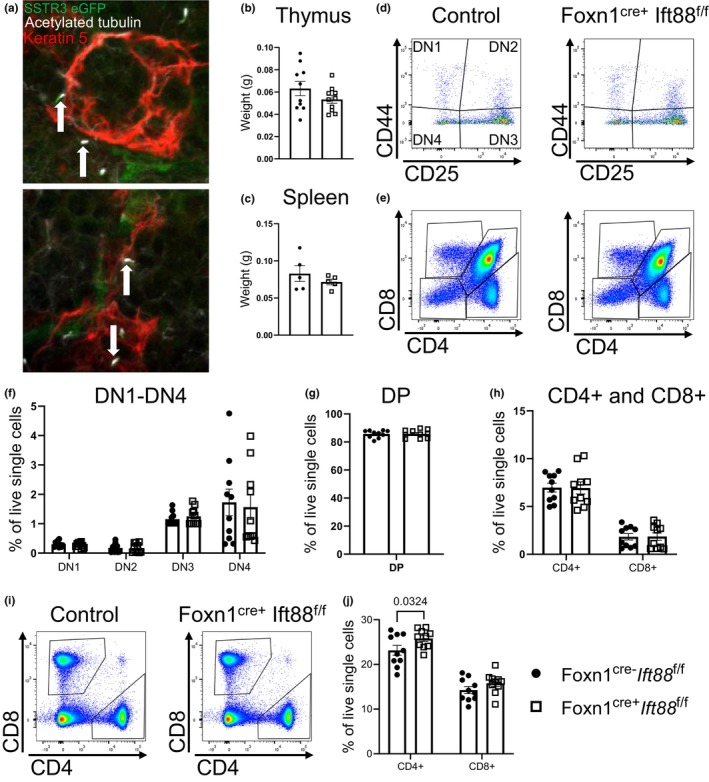
Loss of *Ift88* in thymic epithelial cells does not impact T cell development. (a) Whole mount image of SSTR3:GFP cilia reporter mice co‐stained with acetylated alpha tubulin and keratin 5 using a 60× objective (b, c) Thymus (b) and spleen (c) weight in *Foxn1* ^*cre+*^ *Ift88* ^f/f^ or control mice at 8 weeks of age. (d, e) Representative FACS plots showing DN1‐DN4 thymocytes (d), and CD4+/CD8+ DP, CD4+ SP, and CD8+ SP thymocytes (e) in 8 week old *Foxn1* ^*cre+*^ *Ift88* ^f/f^ or control mice. (f–h) Quantification of DN1‐DN4 thymocytes (f), DP thymocytes (g), and CD4+ and CD8+ SP thymocytes (h) in *Foxn1* ^*cre+*^ *Ift88* ^f/f^ or control mice. (i) Representative FACS plots showing CD4+ and CD8+ T cells in the spleen of *Foxn1* ^*cre+*^ *Ift88* ^f/f^ or control mice at 8 weeks of age. (j) Quantification of CD4+ and CD8+ spleenocytes in *Foxn1* ^*cre+*^ *Ift88* ^f/f^ or control mice. For each quantification (except spleen weight), *N* = 10 mice per group (Controls: 6M, 4F; *Foxn1* ^*cre+*^ *Ift88* ^f/f^: 7M, 3F). Two‐way ANOVA.

Previous data indicate that thymic epithelial cells contain cilia (Bautista et al., 2021; Michelson et al., 2022). To confirm the presence of cilia in the thymus, we performed whole mount imaging of thymic sections obtained from 8‐week‐old somatostatin receptor 3 (SSTR3): GFP reporter mice co‐stained with the cilia marker acetylated alpha tubulin and the thymic epithelial cell marker keratin 5 (Piperno & Fuller, 1985; Rahimi et al., 2021; Trask et al., 1990). SSTR3 is a ciliary localized protein, which allows for visualization of cilia when fused to GFP (Berbari et al., 2008; Handel et al., 1999). Analysis of whole mount images from the thymus indicate strong co‐localization between SSTR3: GFP and acetylated tubulin, with cilia‐like projections protruding from thymic epithelial cells (Figure 1a). We also found cilia‐like projections in cytokeratin 5 negative regions suggesting that cilia are also present on other cell types in the thymus (Figure 1a). To confirm our initial findings, we repeated whole mount imaging using acetylated tubulin and the basal body marker Fibroblast growth factor receptor 1 Oncogene Partner (FOP), in addition to epithelial tight junction marker Zonula Occludens‐1 (ZO1) (Fanning et al., 1998; Lee & Stearns, 2013). Once again, the data indicate that acetylated tubulin and FOP‐positive cilia were found adjacent to ZO1‐positive cells, further confirming the presence of cilia on thymic epithelial cells (Figure S1).

*Ift88* has reported roles in the formation and maintenance of cilia as well as in immune synapse formation (Finetti et al., 2009, 2011). To test the importance of thymic epithelial cell‐expressed *Ift88* and cilia on T cell development, we crossed conditional *Ift88* mice (*Ift88* ^f/f^) to mice expressing a thymic epithelial‐specific cre recombinase (*Foxn1* ^*cre*^) (Blackburn et al., 1996; Gordon et al., 2007). We validated *Foxn1* ^*cre*^ was able to excise the *Ift88* floxed alleles via genotyping (Figure S1). Loss of *Ift88* in thymic epithelial cells did not impact thymus or spleen weight (Figure 1b,c). Next, we analyzed the number of thymocytes using flow cytometry and the gating strategy shown in Figure S3; this gating approach is similar to strategies used in previous flow cytometry studies of the thymus (Lau et al., 2017; Stepanek et al., 2011). Our analysis of flow cytometry data from 8‐week‐old mice indicate that loss of *Ift88* in thymic epithelial cells did not impact the number of DN1‐DN4 thymocytes (Figure 1d,f). Likewise, analysis of CD4+/CD8+ DP thymocytes and CD4+ and CD8+ SP thymocytes showed no difference between groups (Figure 1e,g,h). We also analyzed the impact of thymic epithelial cell *Ift88* deletion on CD4+ and CD8+ T cells in the spleen. The data indicate that mice lacking thymic epithelial cell *Ift88* have significantly more CD4+ T cells, while the number of CD8+ T cells was not different between groups (Figure 1g,h). Collectively, these data indicate that loss of *Ift88* in thymic epithelial cells has minimal impacts on T cell thymic development, although it may impact the number of CD4+ T cells in the periphery.

### 2.2 Loss of *Ift88* in T cells impairs DP to SP thymocyte differentiation

To determine the importance of T cell‐expressed *Ift88* on thymocyte development, we performed flow cytometry analysis on thymocytes isolated from *Ift88* ^f/f^ mice crossed to mice expressing a global, tamoxifen‐inducible cre recombinase (*CaggCre* ^*ERT2*^) and membrane tomato, membrane GFP (mTmG) 3 weeks post tamoxifen induction. In these mice, expression of the cre recombinase fused to the mutated estrogen receptor (cre^ERT2^) is driven by the chicken beta actin promoter (*Cagg*), which is expressed in the majority of cells including T cells (Lohse & Arnold, 1988). When cre activity is induced, the loxp sites surrounding both the membrane tomato and the *Ift88* genes are excised. This results in cells with deletion of both genes, meaning all *Ift88* deficient cells will be GFP+, while *Ift88* wild‐type cells remain TdT+.

**FIGURE 2:**
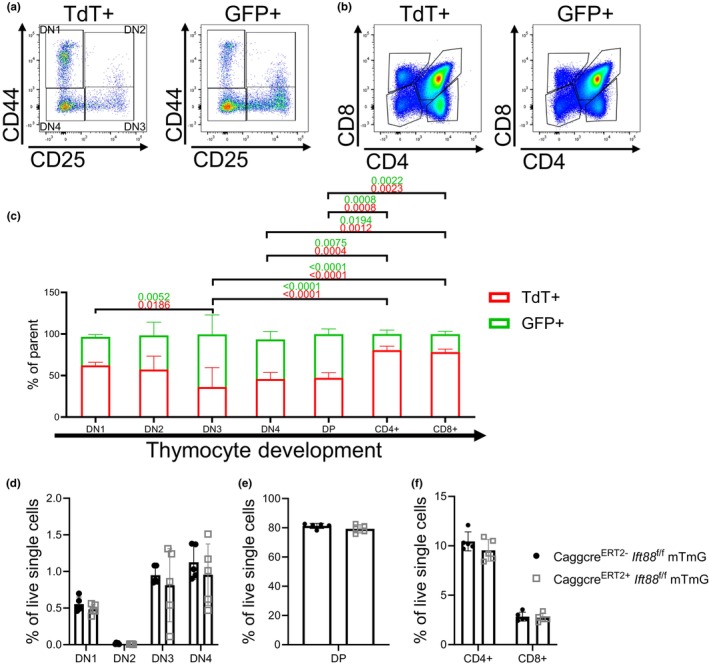
Loss of *Ift88* in T cells prevents DP to SP thymocyte transition. (a, b) FACS plots showing DN1‐DN4 thymocytes (a) and CD4+/CD8+ DP, CD4+ SP, and CD8+ SP thymocytes (b) that are TdT+ (left) and GFP+ (right) 3 weeks post tamoxifen induction in CaggCre^ERT2+^ *Ift88* ^f/f^ mTmG mice. (c) Quantification of the ratio of GFP+ to TdT+ thymocytes during thymocyte development. Data is shown as the percentage of parent cells that express GFP or TdT. (d–f) Quantification of total DN1‐DN4 thymocytes (d), DP thymocytes (e), and CD4+ and CD8+ SP thymocytes (f) in *CaggCre* ^*ERT2+*^ *Ift88* ^f/f^ mTmG or control mice. For each graph, *N* = 5 mice (all male). Two‐way ANOVA.

We first confirmed that an approximately equal ratio of DN1 thymocytes were GFP and TdT positive upon entering the thymus (Figure 2a,c). If *Ift88* was dispensable for T cell development, we would predict that the ratio of GFP+ to TdT+ cells at the DN1 stage would remain constant throughout T cell development and that each stage of development would see no significant changes when compared to the previous stage. Analysis of flow cytometry data indicate that the ratio of GFP+ to TdT+ DN2‐4 and CD4+/CD8+ DP thymocytes was approximately equal compared to their preceding stages, although we did observe an increase in the number of GFP+ T cells between the DN2 to DN3 stage (Figure 2a–c), suggesting that loss of *Ift88* may be enhancing early T cell development. In contrast, the ratio of GFP+ to TdT+ CD4+ and CD8+ SP thymocytes was substantially reduced compared to the DP thymocytes (Figure 2b,c). Despite the significant reduction in the ratio of GFP+ to TdT+ CD4+ and CD8+ SP thymocytes, the total number of DN1‐DN4, CD4+/CD8+ DP, CD4+ SP, and CD8+ SP cells was not different between cre‐positive and cre‐negative mice (Figure 2d–f), suggesting that the TdT+ T cells expressing *Ift88* compensate for the loss of their GFP+ counterparts.

### 2.3 The ratio of GFP+ to TdT+ T cells is drastically reduced in the spleen and kidney

**FIGURE 3:**
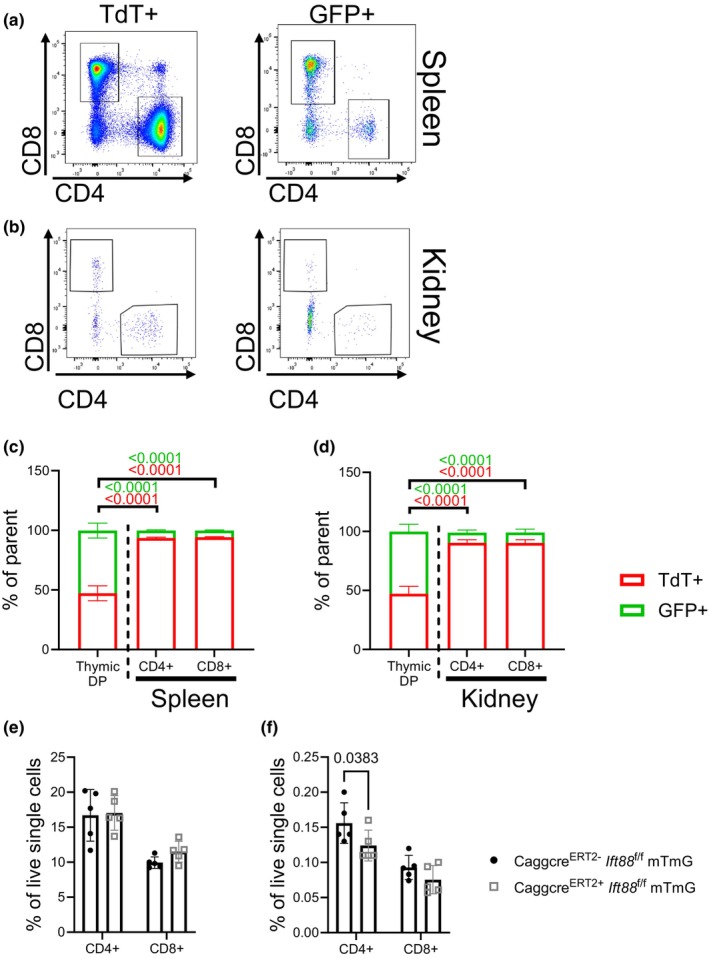
The ratio of GFP+ to TdT+ T cells in the spleen and kidney is substantially reduced. (a, b) FACS plots showing TdT+ and GFP+ CD4+ and CD8+ T cells in the spleen (a) and kidney (b) 3 weeks post‐tamoxifen induction in *CaggCre* ^*ERT2+*^ *Ift88* ^f/f^ mTmG mice. (c, d) Quantification of the ratio of GFP+ to TdT+ cells in the spleen (c) and kidney (d) 3 weeks post‐tamoxifen induction. (e, f) Quantification of total CD4+ and CD8+ T cells in the spleen (e) and kidney (f) 3 weeks post‐tamoxifen induction. *N* = 5 mice (all male). Two‐way ANOVA.

Our data indicate that deletion of *Ift88* in T cells drastically impairs their ability to differentiate into CD4+ or CD8+ SP thymocytes (Figure 2c). To determine the effect of *Ift88* deletion on peripheral T cell accumulation, we analyzed the ratio of GFP+ and TdT+ T cells in the spleen and kidney of *CaggCre* ^*ERT2+*^ *Ift88* ^f/f^ mTmG mice 3 weeks post tamoxifen induction. Our data indicate that the ratio of GFP+ to TdT+ CD4+ and CD8+ T cells in the spleen was significantly reduced compared to the ratio of DP thymocytes (Figure 3a,c). Despite this reduced ratio of GFP+ to TdT+ CD4+ and CD8+ T cells in the spleen, there was no difference in total number of CD4+ or CD8+ T cells in cre‐positive compared to cre‐negative mice (Figure 3e), once again suggesting that T cells with normal *Ift88* expression (TdT+) compensate for the loss of their *Ift88*‐deficient counterparts.

Next, we analyzed the ratio of GFP+ to TdT+ thymocytes in the kidney, as one of the common phenotypes of *Ift88* deletion is cystic kidney disease (Adamiok‐Ostrowska & Piekielko‐Witkowska, 2020; Pazour et al., 2000). Similar to the spleen, the ratio of GFP+ to TdT+ CD4+ and CD8+ T cells in the kidney was drastically reduced compared to DP thymocytes (Figure 3b,d). Interestingly, the total number of CD4+ T cells in the kidney was significantly reduced in cre‐positive mice compared to cre‐negative mice, while the number of CD8+ T cells was not different between groups (Figure 3f).

## 3 DISCUSSION

*Ift88* is involved in cilia formation (Hildebrandt et al., 2011) and has been reported to be present in the immune synapse of T cells (Finetti et al., 2009). In this manuscript, we show that deletion of *Ift88* in thymic epithelial cells has minimal impact on T cell development in 8‐week‐old mice. In contrast, loss of *Ift88* in T cells impairs the transition of CD4+/CD8+ DP thymocytes to CD4+ and CD8+ SP thymocytes as evidenced by the reduced ratio of GFP+ to TdT+ cells during this transition. Loss of *Ift88* in T cells also affected the ratio of GFP+ to TdT+ CD4+ and CD8+ T cells in the spleen and kidney. Despite the reduction of GFP+ CD4+ and CD8+ T cells in the thymus, spleen, and kidney, the total number of CD4+ and CD8+ T cells was minimally impacted in each tissue, likely due to compensation from their TdT+ counterparts. Collectively, these data indicate that T cell‐expressed *Ift88* is critical for CD4+ and CD8+ T cell development and that T cells in CaggCre^ERT2^ *Ift88* ^f/f^ mTmG mice are mostly wild‐type (TdT+) when analyzed 3 weeks post‐tamoxifen injection.

While a previous study reported that thymic epithelial cell‐specific loss of *Ift88* impacted the development of SP thymocytes in mice, it is important to note these differences were only observed in mice 50–85 weeks of age (Kutomi et al., 2022). When the authors analyzed T cell development in mice that were 3–4 weeks of age, they found that loss of thymic epithelial expressed *Ift88* had no impact on T cell development. This is important as data from our study were collected when mice were ~8 weeks of age, a time point much closer to the young mice used in the aforementioned study. It is interesting to note that we did observe increased CD4+ T cells in the spleen of mice lacking thymic epithelial cilia, in line with the previous report. Thus, it is possible that the observed changes found in 50–85‐week‐old mice are already occurring at ~8 weeks of age. A more detailed time course outlining the effects of thymic epithelial‐expressed *Ift88* on T cell development is warranted.

Previous data indicate that *Ift88* forms an immune synapse with *Ift20*, *Ift57*, and *Ift54* to regulate endosomal recycling of the TCR (Finetti et al., 2009). Deletion of *Ift20* using an Lck^cre^, which is active in the DN2 stage of thymocyte development, significantly reduced the number of CD4+ and CD8+ SP thymocytes but had no effect on the number of CD4+/CD8+ DP thymocytes, similar to what we observed in our studies (Carow et al., 2016; Yuan et al., 2014). These data suggest that *Ift20* is critical for transition from the DP to SP stage of thymocyte development. Surprisingly, deletion of *Ift20* using a CD4^cre^, which is active at the CD4+/CD8+ DP thymocyte stage, did not impact CD4+ or CD8+ T cell numbers in the thymus or spleen. These data suggest that either loss of *Ift20* must occur prior to the DP stage or that the CD4^cre^ was unable to fully delete the *Ift20* gene until after the DP to SP transition had occurred. Using our fate mapping approach, we found that *Ift88* expressed in T cells is critical for the successful transition from DP to SP thymocytes. Further, the data support the paradigm that *Ift88* works in concert with *Ift20* to regulate TCR recycling and T cell development, specifically at the DP to SP transition. Whether other IFT genes (*Ift54*, *Ift57*) reported to be a part of this immune complex are required for T cell development remains unknown.

Previous data from our lab indicate that mice lacking *Ift88* (CaggCre^ERT2^ *Ift88* ^f/f^) develop cystic kidney disease within 5–6 months post tamoxifen induction (Zimmerman et al., 2019). Further, we found that kidney injury to these mice 3 weeks after tamoxifen induction resulted in rapidly progressing cystic kidney disease (Aloria et al., 2021; Zimmerman et al., 2019). Loss of adaptive immune cells in this model significantly attenuated the severity of cystic kidney disease after injury (Song et al., 2022). Due to the fact that we used the same global cre recombinase to delete *Ift88* in these studies, and we found that loss of adaptive immune cells, including T cells, significantly attenuated disease, an open question was whether the ability of T cells to promote injury‐accelerated cystic disease was due to intrinsic defects in the T cells or due to how the T cells were interacting with *Ift88*‐deficient epithelium or other cells in the microenvironment. Data presented here shows that the majority of T cells observed in the kidney 3 weeks after tamoxifen induction were *Ift88* wild‐type (TdT+). These data suggest that following tamoxifen induction, GFP+ *Ift88*‐deficient T cells are largely replaced by their TdT+ *Ift88* wild‐type counterparts soon after the DP stage of thymocyte development. The data also suggest that in our previous kidney injury studies, the ability of T cells to accelerate cystic kidney disease is due to their interaction with the *Ift88* deficient epithelium or other cells in the microenvironment. While future studies are needed to fully explain why T cells accelerate cystic disease in the CaggCre^ERT2^ *Ift88* ^f/f^ model, our studies allow us to speculate that it is unlikely that the observed phenotype is due to intrinsic defects present in the T cells.

In sum, our data indicate that loss of *Ift88* in thymic epithelial cells is dispensable for T cell development. In contrast, *Ift88* expression in T cells is critical for the transition from DP to SP thymocytes. Despite this requirement, the total number of CD4+ and CD8+ T cells in CaggCre^ERT2^ *Ift88* ^f/f^ mTmG mice is not different between cre‐positive and cre‐negative mice, likely owing to compensation by *Ift88* proficient T cells.

## 4 METHODS

### 4.1 Mice

*CaggCre* ^*ERT2*^ *Ift88* ^tm1Bky^ (*Ift88* ^f/f^) mTmG C57BL/6J male and female mice were bred in‐house. *Foxn1* ^*cre*^ mice were purchased from Jackson Laboratory (Strain #: 018448) and crossed to *Ift88* ^f/f^ mice. Eight‐ to ten‐week‐old *Caggcre* ^*ERT2*^ *Ift88* ^f/f^ mTmG animals were given an intraperitoneal (IP) injection of tamoxifen at 6 mg/40 g body weight once daily for three consecutive days, similar to our previous report (Zimmerman et al., 2019). Deletion of *Ift88* was confirmed by PCR. Three weeks post tamoxifen injection, mice were anesthetized with Avertin injection at a dose of 375 μg/g of body weight (Fisher Scientific, Catalog#: 421430500); animals were then euthanized by cardiac perfusion and tissues collected for analysis via flow cytometry. *Foxn1* ^cre^ *Ift88* ^f/f^ mice were harvested at 8 weeks of age. All animals were maintained in accordance with NIH, USDA, and AAALAS guidelines and with approval of the Institutional Animal Care and Use Committee (IACUC) of the University of Oklahoma Health Sciences Center under protocol 22‐078‐SACH.

### 4.2 Flow cytometry

Anesthetized mice were perfused with PBS through the left ventricle in order to flush out any immune cells in the vasculature. After perfusion of the mouse with PBS, thymus and spleen were manually disassociated by passage through a 70‐μm mesh filter (Falcon; BD Biosciences) yielding single‐cell suspensions. Kidney tissue was digested in 1 mL of RPMI containing 1 mg/mL collagenase Type I (Catalog#: C0130‐100MG, Sigma‐Aldrich) and 100 U/mL DNase (Catalog#: D5025‐15KU, Sigma‐Aldrich) for 30 min at 37°C. After generating single cell suspensions, red blood cells were lysed using ACK red blood cell lysis buffer (Catalog#: 10128–802, Quality Biological) at room temperature for 5 min. Cells were resuspended in 1 mL of PBS containing 1% BSA with Fc blocking solution for 30 min on ice. 1 × 10^6^ cells were stained for 30 min at room temperature with primary antibody. Primary antibodies were as follows: APC rat anti‐mouse CD25 (Catalog#: 101910, clone 3C7, BioLegend), PE rat anti‐mouse CD3 (Catalog#: 12–0031‐82, clone 145–2 c11, eBioscience), PE Cy7 rat anti‐mouse CD4 (Catalog#: 25–0041‐82, clone GK1.5, Invitrogen), Percpcy5.5 rat anti‐mouse CD8 (Catalog #: 45–0081‐82, clone 53–6.7, Invitrogen), BV786 rat anti‐mouse CD44 (Catalog#: 563736, clone IM7, BD Bioscience), BV605 hamster anti‐mouse TCRβ (Catalog#: 109241, H57‐ APC 597, BioLegend), FITC rat anti‐mouse CD45 (Catalog#: 2023‐08‐08, Clone 30‐F11, ThermoFisher Scientific), and Fixable Aqua Dead Cell Stain (Catalog#: L34957, Invitrogen). Cells were washed with 1% BSA, fixed in 2% PFA for 30 min, and resuspended in PBS. After immunostaining, cells were analyzed on a BD LSRII flow cytometer. Data analysis was performed using FlowJo v10 software.

### 4.3 Whole mount imaging of thymus

Following perfusion of the mouse with PBS, the thymus was isolated and fixed in 4% PFA at 4°C until the tissue sank (2.5 h) followed by washing three times with 1% Triton for 30 min. The tissue was then incubated in blocking solution (PBS, 1% Triton, 1% BSA, 0.2% sodium azide) rocking at 4°C for 2 days. Primary antibodies used include: anti‐acetylated alpha tubulin (Catalog#: T7451, Sigma) direct conjugated to Alexa 647 (Catalog#: A20186, ThermoFisher), ZO‐1 (clone R40.76, used at 1:2), FOP (Fgfr1op) (Catalog#: 11343‐1‐AP, ProteinTech), and Keratin 5 (Catalog#: PRB‐160P‐100, Covance), all diluted in blocking buffer and rocked for 3 days at 4°C. The tissue was washed three times for 10 min each in 1% Triton in PBS. Tissue stained with direct conjugated acetylated tubulin was washed and put into 100% glycerol at 4°C for 2 days. Secondary antibodies for non‐direct conjugate antibodies included the following: Alexa Fluor TRITC‐conjugated anti‐rat (Catalog NO.1834802, Biolegend) and Alexa Fluor FITC‐conjugated anti‐rabbit (Catalog NO: 712–606‐153, Jackson ImmunoResearch). Following addition of secondary antibody, nuclei were stained by Hoechst nuclear stain (Sigma‐Aldrich). All tissues were moved into 75% glycerol before images were captured on a Nikon TI2 Eclipse (Nikon Instruments) spinning disc confocal equipped with a Yokogawa X1 disc (Yokogawa) on an Orca Flash4.0 sCMOS (Hamamatsu) using a 60× APO TIRF 1.49NA (Nikon Instruments) objective. Confocal images were processed and analyzed in NIS Elements software.

### 4.4 Statistics and data analysis

Data is presented as mean ± SD. Significant differences were determined in one of two ways: unpaired student *t* tests were used for head‐to‐head comparisons, while two‐way ANOVAs were used to compare multiple groups. Data was considered statistically significant for *p* values less than 0.05. All statistical testing was performed using GraphPad Prism, version 10.1.0 (316).

## AUTHOR CONTRIBUTIONS

S.M., N. G., B.Y., and K.Z. were responsible for conceptualization. Studies and experiments were performed by S.M., N.G., M.S., and M.C. Analysis and writing was done by S.M., N.G., and K.Z. Funding was acquired for the project by S.M., K.Z., and B.Y.

## CONFLICT OF INTEREST STATEMENT

None of the authors has any affiliation or involvement with any entity with financial or non‐financial interest in the research discussed in this manuscript.

## ETHICS STATEMENT

All animals were maintained in accordance with NIH, USDA, and AAALAS guidelines, and with approval of the Institutional Animal Care and Use Committee (IACUC) of the University of Oklahoma Health Sciences Center under protocol 22‐078‐SACH.

## Supporting information


Figure S1.

## Data Availability

Data will be made available upon reasonable request.
