# Supplementary material for: T cell‐expressed Ift88 is required for proper thymocyte differentiation in mice
Source: Physiol Rep. 2024 Nov 19;12(22):e70120. doi: 10.14814/phy2.70120 (PMC11576126; doi:10.14814/phy2.70120)
Supplement: Supplementary file 1 — Figure S1. [file PHY2-12-e70120-s001.zip › Figure Captions.docx]

**Figure S1.** Immunofluorescence staining of cilia in adult mouse thymus. Whole mount image of mouse thymus co-stained with acetylated alpha tubulin, the cilia basal body marker FOP, and epithelial tight junction marker ZO1 taken using a 60x objective.

**Figure S2. Genotypes of control and Foxn1^cre^ *Ift88*^f/f^ mice at 8** **weeks of age.** Representative genotyping gel depicting expression of the *Ift88* floxed allele and the *Ift88* delta band in DNA that was extracted from the ears of control or *Foxn1^cre+^* *Ift88*^f/f^ mice at 8 weeks of age. Importantly, *Foxn1* is expressed by epithelial cells in the ear (Blackburn et al., 1996; Romano et al., 2013).

**Figure S3.** Gating strategy for used to identify thymocytes. Gating strategy used during flow cytometry to identify immune cell populations, including CD4+, and CD8+ cells, and DN1-DN4 thymocyte subsets. Antibodies used can be found in Flow Cytometry section of Methods.
