# Supplementary figures and images for: T cell‐expressed Ift88 is required for proper thymocyte differentiation in mice
Source: Physiol Rep. 2024 Nov 19;12(22):e70120. doi: 10.14814/phy2.70120 (PMC11576126; doi:10.14814/phy2.70120)

Supplemental figure 1

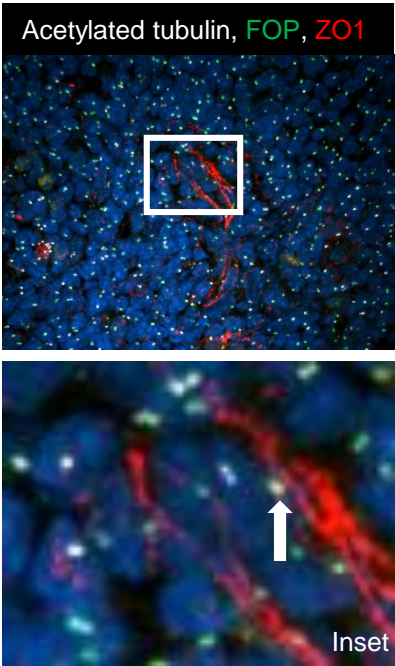

Supplement: Supplementary file 1 — Figure S1. [file PHY2-12-e70120-s001.zip › PHYSREP-2024-07-449-f04-z-.pdf]

Supplemental figure 2

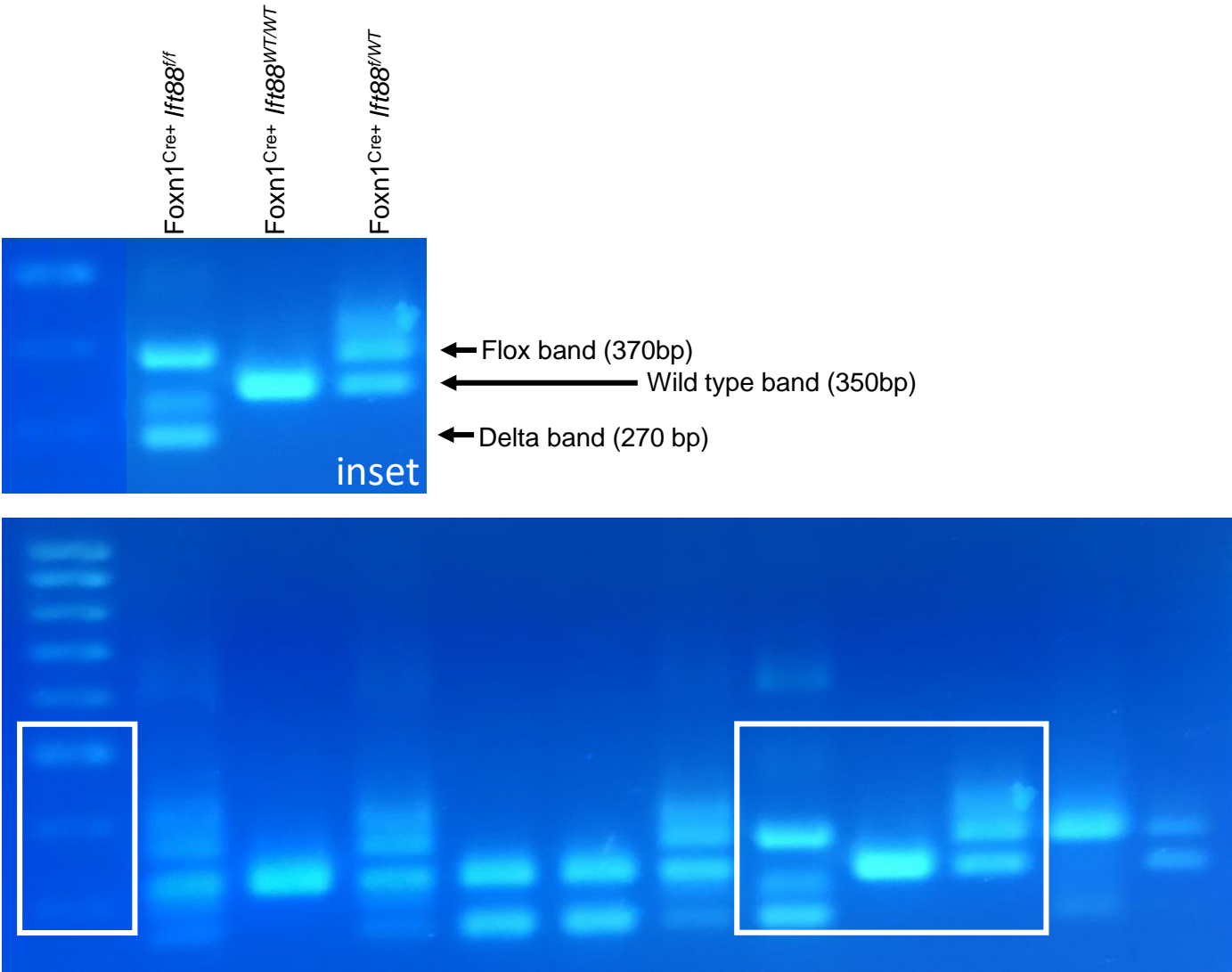

Supplement: Supplementary file 1 — Figure S1. [file PHY2-12-e70120-s001.zip › PHYSREP-2024-07-449-f05-z-.pdf]

Supplemental figure 3

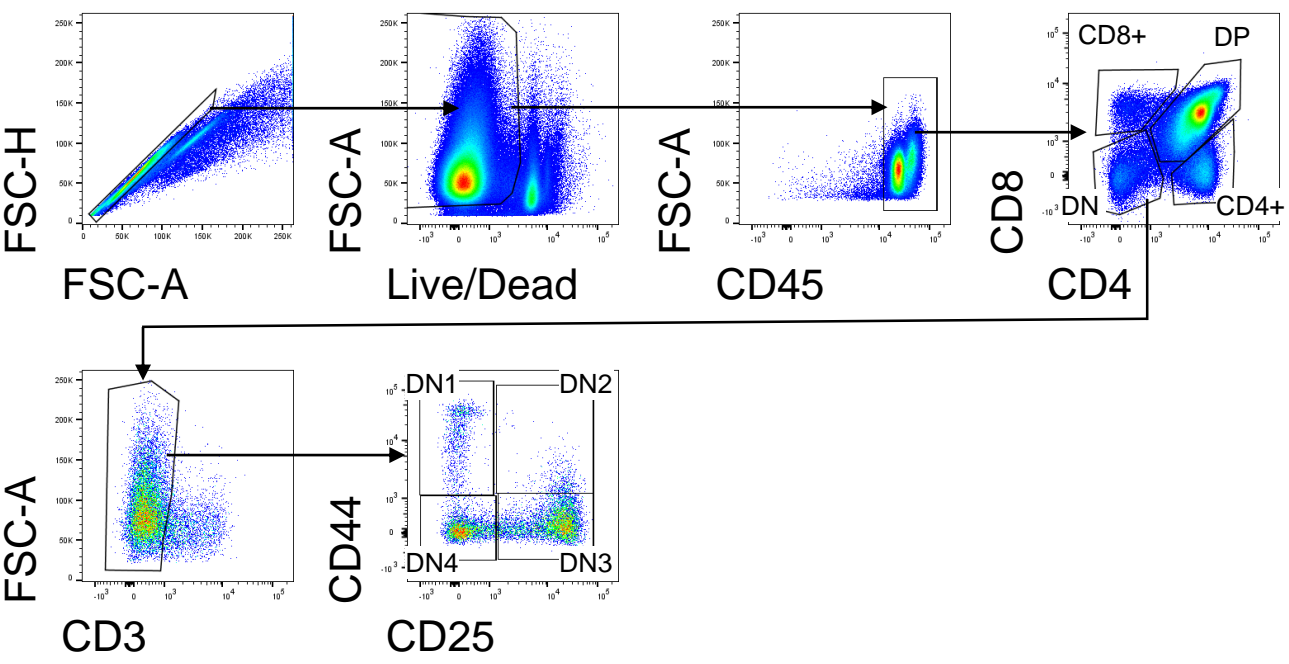

Supplement: Supplementary file 1 — Figure S1. [file PHY2-12-e70120-s001.zip › PHYSREP-2024-07-449-f06-z-.pdf]
